# Research Verification in the Application Review Process for Orthopaedic Surgery Residency

**DOI:** 10.7759/cureus.81511

**Published:** 2025-03-31

**Authors:** Rithvik Vutukuri, Ryan C White, Shreya M Saraf, Mary K Mulcahey

**Affiliations:** 1 Orthopaedic Surgery, Tulane University School of Medicine, New Orleans, USA; 2 Orthopaedic Surgery, Stritch School of Medicine, Loyola University, Chicago, USA; 3 Orthopaedic Surgery, Loyola University Medical Center, Chicago, USA

**Keywords:** orthopaedic surgery residency, program director survey, research misrepresentation, research verification, residency applications

## Abstract

Introduction: The integrity of applications submitted by medical students applying for orthopaedic surgery residency has been a topic of concern within the medical community in recent years. Although research misrepresentation is a recognized issue, there is limited information on how orthopaedic surgery residency programs verify the research contributions listed by medical students during the application process. The purpose of this study is to identify if and how orthopaedic surgery residency programs verify the research section of an application.

Methods: A 28-question anonymous survey was distributed in March 2024 to program directors, assistant program directors, and research directors in the department of orthopaedic surgery at participating Collaborative Orthopaedic Educational Research Group (COERG) programs. The survey was open for three weeks and assessed the respondents’ background, their application review practices, and the reasoning behind these practices.

Results: There were a total of 10 respondents: eight identified as male (80%), one identified as female (10%), and one identified as a transgender female (10%). Six of 10 (60%) respondents did not verify research listed on orthopaedic surgery residency applications. Only three (30%) verified both posters and publications, and one (10%) participant verified publications only. Of those who verified research, the most common verification process was both checking PubMed/similar platforms (three, 33%) and contacting research mentors or collaborators (three, 33%). Six (60%) respondents were at least somewhat concerned with research misrepresentation, and eight (80%) felt that verification of research on Electronic Residency Application Service (ERAS) applications should be standard practice. The primary reason cited by programs that did not verify research was the significant time investment required to establish a consistent process, accounting for eight (47%) responses. Four (40%) respondents felt that incorporating research verification into the application review process would extend each review by 31 minutes to an hour.

Conclusion: Orthopaedic surgery residency programs are generally concerned with research misrepresentation among applicants; however, most programs are not yet actively verifying research reported in the ERAS application. The primary concern among programs is the time required for verification. There is a need for the creation of standardized research verification practices that can be adopted by all programs. Programs may consider implementing more efficient verification strategies or prioritizing confirmation of an applicant’s most significant research achievements to ensure a fair and accurate evaluation of applicants.

## Introduction

Orthopaedic surgery continues to be one of the most competitive medical specialties to match into. With 1,492 applicants applying for 916 available positions through the Electronic Residency Application Service (ERAS) in the 2023-2024 National Resident Matching Program (NRMP) match cycle, there was a match rate of 61%, leaving 575 students unmatched [1]. As Step 1 has transitioned to pass/fail, students have been encouraged to pursue more research in order to strengthen their residency application [2]. Unfortunately, as research activity per applicant increases and known issues of research misrepresentation persist, concerns within the medical community regarding the integrity of the application process for orthopaedic surgery residency programs have heightened.

Several previous studies have investigated research misrepresentation by orthopaedic surgery residency applicants [3-8]. Through three consecutive retrospective verification studies conducted by Crosby et al., research misrepresentation in orthopaedic surgery residency applications has been longitudinally well documented [3,7,8]. These studies included application reviews during the 1998-1999, 2005-2006, and 2016-2017 match cycles. The authors defined misrepresentation as stating false authorship for a study, stating authorship of a non-existent article, or promoting themselves to a higher authorship status. Crosby et al. found that among orthopaedic surgery residency applicants to their program, 18% of publications listed in applications were misrepresented during the 1998-1999 cycle [3]. This rate increased to 20.6% in the 2005-2006 cycle [7]. However, by the 2016-2017 cycle, only 1.18% of citations in applications to their program were found to be misrepresented [8]. The authors hypothesized that this trend could be attributed to the addition of the PubMed Identifier (PMID) to ERAS applications.

A 2019 retrospective study by El Beaino et al. examined the incidence of research misrepresentation among applicants to the University of Texas Galveston's 2017 orthopaedic surgery residency program [6]. Misrepresentation was defined as altering or adding a candidate's name in the authorship order or listing non-peer-reviewed publications under the peer-reviewed section in ERAS. Using PubMed (http://www.ncbi.nlm.nih.gov/pubmed/) and Google Scholar (https://scholar.google.com/) to verify published, accepted, or in-press papers, the authors identified a 20.5% incidence of misrepresentation among applicants. Most recently, a 2022 systematic review by Burkhart et al. found that nearly 20% of applications to orthopaedic surgery residency and fellowship programs contain at least one form of misrepresentation of research [4]. The types of misrepresentation included non-authorship of an existing article, claimed authorship of a non-existent article, or incorrect listing of authorship order for an existing article, the most common of which was found to be the authorship of a non-existent article. Unfortunately, misrepresentation has been documented in many other fields as well [9-15]. While some of these inaccuracies may not be intentional, the prevalence of this issue is concerning and necessitates further exploration.

There is a paucity of literature evaluating the verification of research during the orthopaedic surgery residency application review process. To our knowledge, there are no previous studies that have explored whether programs verify the research listed on ERAS applications. The purpose of this study was to identify if and how orthopaedic surgery residency programs verify the research section of an application.

## Materials and methods

Cross-sectional survey construction

An anonymous 28-question cross-sectional survey was created using Microsoft Forms (Microsoft Corp., Redmond, WA, US) and distributed to 10 orthopaedic surgery residency program directors, assistant program directors, and research directors at participating Collaborative Orthopaedic Educational Research Group (COERG) programs. These 10 individuals indicated a willingness to receive the survey after this project was presented to COERG. The COERG, whose mission is to support the orthopaedic surgery training community by enhancing resident education and faculty development research, fosters collaboration among these leaders to improve the overall quality of training programs. The survey included questions related to demographics, which positions respondents held in their respective institutions, thoughts regarding research misrepresentation by orthopaedic surgery residency applicants, current application review protocols, and thoughts on research verification/misrepresentation in general. The survey can be found in the appendix.

Survey dissemination

The anonymous survey was distributed in March 2024 to 10 orthopaedic surgery residency program leaders who are members of COERG and expressed interest in participating in this study. The survey was active for three weeks.

Data collection and statistical analysis

Survey responses were exported into Microsoft Excel (Microsoft Corp., Redmond, WA, US) where descriptive analysis and graphic representations were used to report and assess survey responses, trends, and research findings. Descriptive techniques were used to summarize the responses. Multiple-choice questions were presented using frequency distributions and percentages. Open-ended responses were categorized into common themes for qualitative analysis. Data was visually represented using graphs and tables to illustrate trends. Anonymous survey responses and demographic data were entered into a data collection tool and stored in a password-protected database. To protect the privacy of participants, survey questions only asked general information about active research verification and did not ask for personally identifiable information or pertain to any sensitive subject matter.

## Results

Demographics of respondents

There were 10 respondents: seven (70%) program directors, two (20%) assistant program directors, and one (10%) research director. Of the 10 respondents, eight (80%) were male, one (10%) was female, and one (10%) was a transgender female. A majority of participants (six, 60%) had been in their current position for 0-4 years, while the remainder (four, 40%) had been in their roles for 5-9 years. Five (50%) respondents were from the Northeast, one (10%) was from the Midwest, and two (20%) were from either the South or the West. The full breakdown is shown in Table 1.

**Table 1:** Demographics of Respondents

Gender	n (%)
Male	8 (80)
Female	1 (10)
Transgender female	1 (10)
Position	
Program director	7 (70)
Assistant program director	2 (20)
Research director	1 (10)
Years in current position	
0-4 years	6 (60)
5-9 years	4 (40)
Geographic region	
Northeast	5 (50)
Midwest	1 (10)
South	2 (20)
West	2 (20)
Program type	
Academic-based	6 (60)
Community-based	3 (30)
Other	1 (10)

Research verification and methods

When asked about current ERAS research verification processes, three (30%) respondents reported that they verify both publications and posters, while one (10%) verifies only publications, and six (60%) do not verify research at all. Among those who conduct verification, a variety of methods were employed: three (33%) respondents use PubMed or similar databases, another three (33%) communicate with collaborators or research mentors, one (11%) requests documentation directly, and two (22%) utilize other verification techniques.

Concern regarding research misrepresentation

**Figure 1:**
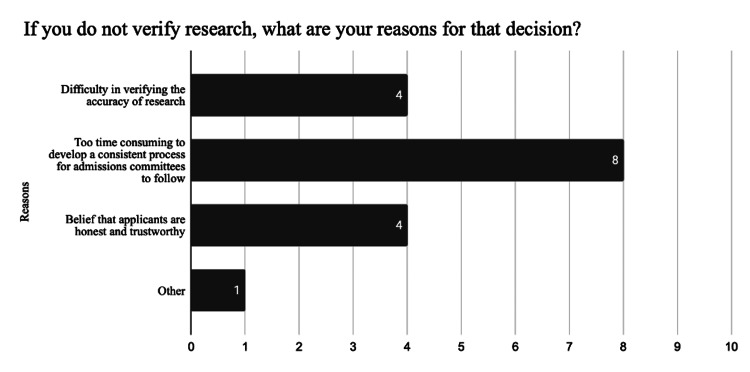
Reasons for Not Verifying Research

**Figure 2:**
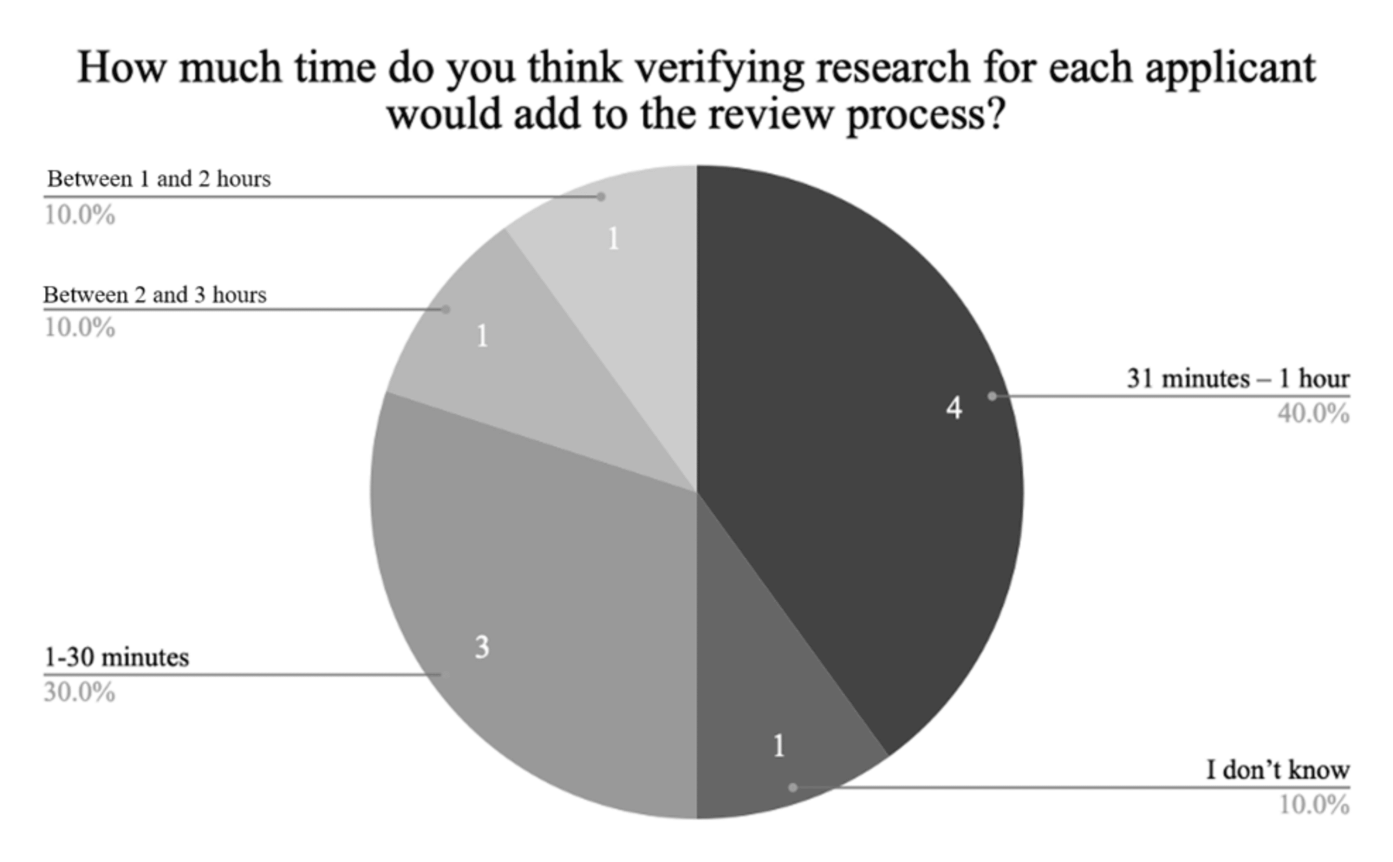
Additional Time Required to Verify Research

Respondents were queried regarding their concern about the prevalence of research misrepresentation in orthopaedic surgery residency applications. Four (40%) respondents indicated they were not very concerned, two (20%) were somewhat concerned, three (30%) expressed concern, and one (10%) was very concerned. In exploring why some participants do not verify research, we found that eight (47%) considered it too time-consuming, four (23.5%) noted the difficulty of verification, and another four (23.5%) trusted in the honesty of students, seeing no need for verification (Figure 1). In terms of the additional time needed for research verification of each application, responses varied: one (10%) estimated it would add 2-3 hours, one (10%) thought it would add 1-2 hours, four (40%) anticipated an additional 31 minutes to one hour, and three (30%) expected it to take an extra 1-30 minutes (Figure 2).

Research verification as a goal

Out of the 10 participants, eight (80%) believed that reported research should be verified. However, the same number of participants (eight, 80%) do not prioritize research verification as part of their interview goals.

## Discussion

The purpose of this study was to identify if and how orthopaedic surgery residency programs verify the research section of an application. Our survey of orthopaedic residency program leaders revealed that while most respondents believe research should be verified, less than half currently engage in any form of verification. Among those who do verify, methods include PubMed searches, communication with collaborators, and documentation requests being the most common approaches. Despite the recognition of the importance of research verification, a significant barrier is the perceived time burden, with most respondents estimating that it would add 31 minutes to an hour per application review. As a result, over half of respondents do not verify research, citing time constraints, difficulty in verification, or trust in applicant honesty as reasons.

While our findings support the series of studies conducted by Crosby et al. in recognizing the importance of research verification, they also reveal a notable gap between recognizing the issue and taking action [3,7,8]. Though Crosby et al. found that a verification process like requiring a PMID correlates with a decrease in misrepresentation, the present study indicates that many programs continue to notice misrepresentation in applications and struggle with implementing other verification practices, primarily due to time constraints [3].

Many studies did not observe a decrease in misrepresentation, including a systematic review by Burkhart et al. that evaluated research misrepresentation by orthopaedic surgery residency and fellowship applicants from 1996 to 2019 [4]. The authors found that nearly 20% of orthopaedic surgery residency and fellowship applications included some form of research misrepresentation. The present study furthers Burkhart et al.’s findings by offering an explanation for why verification remains inconsistent despite broad acknowledgment of the problem. Currently, verification approaches include checking PubMed, Google Scholar, and corresponding journals' websites [6]. While this only allows the confirmation of publications listed in either ‘peer-reviewed journal article/abstracts’, ‘peer-reviewed online publications’, or ‘peer-reviewed journal article/abstracts (other than published)’ parts of the applications, the results of this study suggest that this could serve as an initial step towards mitigating the issue of research misrepresentation. However, the wide array of verification processes available demonstrates the absence of an accepted standardized approach. As Burkhart et al. advise, this lack of verification standardization should be addressed by governing bodies [4].

Most respondents do not consider research verification a goal during interviews, underscoring the belief that verification should precede this stage. This approach allows for interviews to be conducted with full confidence in the accuracy of applications, thereby focusing attention on the applicants themselves. However, to fully address this issue, it is essential to develop standardized verification methods across programs. Residency programs could benefit from collaborative efforts to develop standardized verification protocols. By implementing these measures, the goal is to deter future instances of misrepresentation and maintain the integrity of the residency selection process.

Limitations

There are several limitations to this study. First, there was a small sample size; however, given the geographic diversity of our respondents, the results provide a general overview of the sentiment regarding research verification. Regardless, it calls for further investigation with a larger cohort for more definitive conclusions. Furthermore, every respondent might not be aware of the prevalence of research misrepresentation. Next, as is common with most survey designs, there is a potential for response bias. Finally, as much of our survey was fixed multiple-choice responses, participants may have felt limited in their answers.

## Conclusions

Orthopaedic surgery residency programs are generally concerned with research misrepresentation among applicants; however, most programs are not yet actively verifying research reported in the ERAS application. The primary concern among programs is the time required for verification. There is a need for the creation of standardized research verification practices that can be adopted by all programs. Programs may consider implementing more efficient verification strategies or prioritizing confirmation of an applicant’s most significant research achievements to ensure a fair and accurate evaluation of applicants.

## Appendices

**Table 2:** Distributed Survey ERAS: Electronic Residency Application Service; USMLE: United States Medical Licensing Examination Credits: Rithvik Vutukuri, Ryan C. White, Shreya M. Saraf, and Mary K. Mulcahey

Question	Response options
What is your position/title?	Program Director, Assistant Program Director, Other - Please specify
How would you describe your program?	Academic-based practice, Community-based practice, Other – Please specify
Which region is your residency program located in?	Northeast: CT, ME, MA, NH, NJ, NY, PA, RI, VT; Midwest: IL, IN, IA, KS, MI, MN, MO, NE, ND, OH, SD, WI; South: AL, AR, DE, D.C., FL, GA, KY, LA, MD, MS, NC, OK, SC, TN, TX, VA, WV; West: AK, AZ, CA, CO, HI, ID, MT, NV, NM, OR, UT, WA, WY
What is your gender identity?	Male, Female, Transgender male, Transgender female, Gender binary non-conforming, Other – Please specify
How long have you been in your current position within the orthopaedic surgery department?	0-4 years, 5-9 years, 10-14 years, 15-19 years, 20-24 years, 25-29 years, >30 years
An applicant’s total number of publications is important for them to be considered competitive	Not considered, Disagree, Neutral, Somewhat agree, Strongly agree
What is the minimum # of publications to be considered competitive in your program?	No minimum/0, 1, 2-3, 4-5, 5-9, 10+
The impact factor of the journal of publication is important	Not considered, Disagree, Neutral, Somewhat agree, Strongly agree
The authorship position within the publication is important	Not considered, Disagree, Neutral, Somewhat agree, Strongly agree
The type of study (prospective vs. retrospective vs. case report) is important	Not considered, Disagree, Neutral, Somewhat agree, Strongly agree
Please order these items from most highly regarded (top) to least highly regarded (bottom)	Educational study (in Ortho), Clinical research study (in Ortho), Clinical research study (Non-Ortho), Basic science study, Case report
The type of conference (local/regional/national) is important	Not considered, Disagree, Neutral, Somewhat agree, Strongly agree
Whether the applicant was the presenting author at the conference is important	Not considered, Disagree, Neutral, Somewhat agree, Strongly agree
Do you consider podium presentations more favorably than poster presentations?	Yes, Strongly, Somewhat, No, No difference, Not considered
Research performed before medical school is considered equal to that performed during medical school	Not considered, Disagree, Neutral, Somewhat agree, Strongly agree
Research has become more important when reviewing applications following USMLE Step 1 moving to pass/fail	Not considered, Disagree, Neutral, Somewhat agree, Strongly agree
Applicants that take a research year are more competitive to match at my program	Not considered, Disagree, Neutral, Somewhat agree, Strongly agree
How much time do you typically spend evaluating the research section of a medical student's application?	<1 min, 1-3 mins, 3-5 mins, 5-10 mins, 10-15mins, 15+ mins
Does your orthopaedic surgery residency program verify research (publications, posters, podium presentations, abstracts) reported on ERAS applications?	Yes - publications, Yes - posters, podium presentations, Yes - both publications and posters/podium presentations, No
If your program does verify research, what methods do you use to verify the information provided by applicants?	Checking PubMed (or similar platform) for published articles or abstracts, Contacting research mentors or collaborators, Requesting additional documentation from applicants (e.g., research posters, presentations, and abstracts)
If your program does not verify research, what are the reasons for this decision?	Lack of time and resources, Difficulty in verifying the accuracy of research, Belief that applicants are honest and trustworthy, Too time-consuming to develop a consistent process for admissions committees to follow
How concerned is your program about the prevalence of research misrepresentation in orthopaedic surgery residency applications?	Very concerned, Somewhat concerned, Concerned, Not very concerned, Not at all concerned
Do you believe that the verification of research reported on ERAS applications should be a standard practice in orthopaedic surgery residency admissions?	Yes, No, Not sure
Is research verification one of your goals during the interview process?	Yes, No, Not sure
How much time do you think verifying research for each applicant would add to the review process?	1-30 mins, 31 mins-1 hour, Between 1 and 2 hours, Between 2 and 3 hours, I don’t know
How would you summarize the role research plays in the ranking of an applicant?	Free text response
When examining an applicant’s research, are there any common red flags you notice or things you see that negatively impact your ranking of an applicant?	Free text response
Any other comments regarding your views on research for medical students applying to orthopaedic surgery residency?	Free text response
